# Meroterpenoid-Rich Ethanoic Extract of *Sargassum macrocarpum* Ameliorates Dextran Sulfate Sodium-Induced Colitis in Mice

**DOI:** 10.3390/foods11030329

**Published:** 2022-01-25

**Authors:** Eun-Ji Joung, Lei Cao, Wi-Gyeong Gwon, Mi-Sung Kwon, Kwon Taek Lim, Hyeung-Rak Kim

**Affiliations:** 1 Department of Food Science and Nutrition, Pukyong National University, Busan 608737, Korea; eunji2007@naver.com (E.-J.J.); kwk0113@naver.com (W.-G.G.); mskwon80@hanmail.net (M.-S.K.); 2 Institute of Marine Sciences, Pukyong National University, Busan 608737, Korea; caolei0630@pknu.ac.kr; 3 Department of Display Engineering, Pukyong National University, Busan 608737, Korea; ktlim@pknu.ac.kr; 4 Department of Smart Green Technology Engineering, Pukyong National University, Busan 608737, Korea

**Keywords:** *Sargassum macrocarpum*, inflammatory bowel disease, colitis, anti-inflammation, dextran sulfate sodium, NF-κΒ

## Abstract

Colitis is a colon mucosal disorder characterized by intestinal damage and inflammation. This current study aimed to evaluate the effect of meroterpenoid-rich ethanoic extract of a brown algae, *Sargassum macrocarpum* (MES) on dextran sulfate sodium (DSS)-induced colitis in mice and explore the possible mechanisms. Mice were given 4% DSS in drinking water for 7 days to induce colitis, followed by 3 days of regular water. MES (12 mg/kg body weight) or celecoxib (10 mg/kg body weight) was administrated orally to mice on a daily basis during these 10 days. Both MES and celecoxib supplementations significantly attenuated DSS-induced weight loss, shortening of colon length, elevated myeloperoxidase activity as well as histomorphological changes of colon. MES and celecoxib reduced the inflammation level of colon tissue, as indicated by its suppression on a panel of pro-inflammatory cytokines, including interleukin (IL)-1β, IL-17, tumor necrosis factor α, and interferon γ, and a group of inflammatory proteins, including intracellular adhesion molecule 1, vascular adhesion molecule 1, matrix metalloproteinase (MMP)-2, MMP-9, MMP-13, and inducible nitric oxidase. In addition, their administration down-regulated pro-inflammatory cytokines in serum. Moreover, the supplementation of MES suppressed the DSS-induced hyperactivation of Akt, JNK, and NF-κB signaling pathways. Taken together, our results demonstrate that MES ameliorates DSS-induced colitis in mice, suggesting that MES may have therapeutic implications for the treatment of colitis.

## 1. Introduction

Inflammatory bowel disease (IBD), which includes Crohn’s disease (CD) and ulcerative colitis (UC), is a chronic and recurrent gastrointestinal disorder characterized by recruitment and undesired retention of immune cells in inflamed digestive track. Symptoms include weight loss, fever, diarrhea and bloody stools [1,2]. IBD affects millions worldwide and its prevalence increases in Asian countries, such as China and South Korea [3,4,5]. The precise etiology of IBD and underlying mechanisms remain elusive. However, many studies have shown that causes of IBD involve environmental, individual genetic susceptibility, infection, and immunological factors [6,7]. Currently, therapeutic agents for IBD includes TNF-α antagonists, aminosalicylates, steroids, and immunomodulators [8]. However, long-term use of these drugs have adverse effects, such as gastrointestinal intolerance, hepatotoxicity and increased malignancies [9]. Effective and safe products, especially with natural origin, for the suppression of IBD are needed.

The progress of IBD involve a variety of inflammatory-related proteins. For instance, activation of immune cells elevates the release of pro-inflammatory cytokines and chemokines, such as TNF-α, interleukin-1β (IL-1β), and IL-6, that drive the inflammatory process which leads to tissue damage [10]. Adhesion molecules, such as intercellular adhesion molecule-1 (ICAM-1) and vascular cell adhesion molecule-1 (VCAM-1), expressed by leucocytes and endothelial cells, modulate the recruitment of circulating immune cells to the inflamed intestine [11]. Proteolytic activities of matrix metalloproteinases (MMPs), as extracellular matrix degrading endopeptidases, including MMP-2, MMP-9 (gelatinases) and MMP-13 (collagenases), are associated with tissue-destruction and damage [12].

Seaweed has been utilized as food, medicines, fertilizer, and animal feed for centuries [13]. Seaweed shows the ability to ameliorate digestive problems, reduce cancer occurrence, obesity, and cardiovascular diseases [14,15]. With its health benefits being recognized, its consumption is growing. A group of seaweed-derived substances has exhibited protective effects against colitis-induced colon damages, such as aqueous extract of *Laminaria japonica* [16], a mineral extract from *Lithothamnion corallioides* [17], and fucoidan from *Fucus vesiculosus* [18]. *Sargassum macrocarpum*, a brown alga, is a rich source of anti-inflammatory compounds [19,20,21,22]. In previous studies, we have found a strong anti-inflammatory activity of meroterpenoid-rich ethanoic extract of *S. macrocarpum* (MES) using macrophages, human umbilical vein endothelial cells, and collagen-induced rheumatoid arthritis mice [21,23,24]. It also shows regulation on critical inflammatory response signaling pathways, such as MAPK and NF-κB [24,25]. These two pathways strongly influence the course of UC [24,26].

Dextran sulfate sodium(DSS)-induced colitis is a widely used animal model of colonic inflammation that resemble several features of UC. Therefore, in this study, we investigated the effects of MES supplementation on intestine condition, inflammatory cytokines, inflammation regulation pathways, using DSS-treated mice.

## 2. Materials and Methods

### 2.1. Reagents

Primary and secondary antibodies were obtained from Santa Cruz Biotechnology (Santa Cruz, CA, USA) and Abcam (Danvers, MA, USA). Enhanced chemiluminescence (ECL) detection kit was purchase from GE Healthcare Bio-Sciences (Piscataway, NJ, USA). Enzyme-linked immunosorbent assay (ELISA) kits and Proteome profiler array kit (Mouse XL Cytokine Array Kit) were obtained from R&D System (Minneapolis, MN, USA). Dextran sulfate sodium (DSS, molecular weight 36–50 kDa) was obtained from MP Biomedicals Inc. (Irvine, CA, USA).

### 2.2. Preparation of MES and Analysis of Chemical Components

*S. macrocarpum* was harvested along the coast of Busan, South Korea, in May 2015. The specimen was identified by an algal taxonomist (C.G. Choi), at the Department of Ecological Engineering, Pukyong National University, South Korea. Voucher specimen was deposited in Dr. Choi’s laboratory (CCG51814). The fresh material of *S. macrocarpum* was air dried and stored in a deep-freezer until subsequent extraction. The extract preparation and active component analysis have been described previously [24]. In brief, dried sample (1.0 kg) was extracted twice with 70% (*v*/*v*) ethyl alcohol (5 L each) for 3 h each at 70 °C. The ethanol extract was filtered through an ultrafiltration system (MWCO, 50 kDa) and evaporated ethanol with vacuum evaporator (Eyela N3010, Tokyo, Japan) until the lipid compound was separated from the water fraction. The separated lipophilic fraction was washed with distilled water twice and concentrated by a vacuum evaporator at 45 °C until water content was less than 5% (*w*/*w*). The combined extracts were concentrated and lyophilized by a vacuum evaporator at 45 °C to obtain the MES (80 g). The isolated compounds from MES were identified as sargahydroquinoic acid (SHQA) (37.6%), sargachromeol (SCM) (6.23%), and sargaquinoic acid (SQA) (1.89%), according to our previous study [27].

### 2.3. Animals and Experimental Design

**Figure 1:**
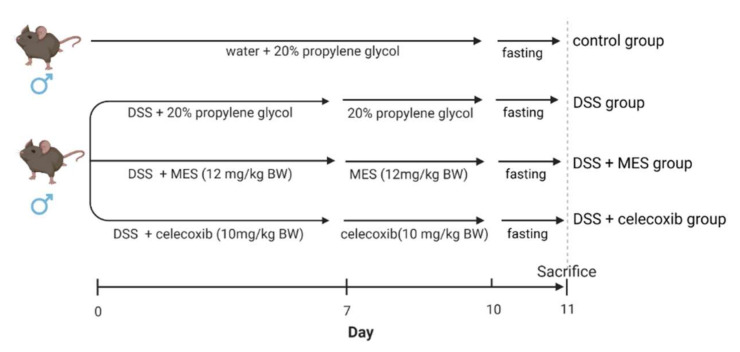
Experimental design. Mice were given 4% DSS in drinking water for 7 days to induce colitis. MES (12 mg/kg BW) or celecoxib (10 mg/kg BW) was given for 10 days. DSS: dextran sulfate sodium; MES: meroterpenoid-rich ethanoic extract of a brown algae, *Sargassum macrocarpum*.

All mouse studies were accomplished with the guidelines of the Korean National Institute of Health Guide for the Care and Use of Laboratory Animals, and were approved by the Animal Ethics Committee of Pukyong National University. Six week-old BALB/c male mice were purchased from Central Lab Animal Inc. (Gyeonggido, Korea). After one week of acclimation, mice (*n* = 8 per group) were randomly assigned to four groups: (1) control, (2) DSS, (3) DSS + MES (12 mg/kg body weight), and (4) DSS + celecoxib (10 mg/kg body weight) (Figure 1). Colitis was induced through drinking water containing 4% (*w*/*v*) DSS. The mice received either regular drinking water (control group) or 4% DSS drinking water (the other three groups) for 7 days and thereafter all mice were provided with regular drinking water for 3 days. Celecoxib, a cyclooxygenase-2 (COX-2) selective inhibitor, significantly ameliorated colitis in various animal models, and therefore was used as a positive control [28,29,30,31]. All the mice were fed with an AIN-93G purified diet. During the 10-day course, the DSS + MES group mice were given MES (12 mg/kg BW), and DSS + celecoxib group mice were given celecoxib (10 mg/kg BW) via oral gavage on a daily basis. Control and DSS groups were given an equal volume of 20% propylene glycol. After one day of fasting, all mice were sacrificed by cervical dislocation. The intake amount of MES was determined based on our previous study, which observed a strong anti-inflammatory effect of both 12 mg MES/kg BW and 24 mg MES/kg BW on lipopolysaccharide-stimulated mice [32]. The difference between 12 mg MES/kg BW and 24 mg MES/kg BW was not significant. Therefore, we hypothesized that 12 mg/kg MES may exert anti-inflammatory effect in DSS-induced colitis mice.

### 2.4. Dosage Information

The human equivalent dose (HED) is determined by the following equation: HED (mg/kg) = Animal dose (mg/kg) × Km ratio [33]. Km ratio was taken as 0.081 from mouse to human. Therefore, HES = 12 mg/kg × 0.081 = 0.97 mg/kg. MES up to 100 mg/kg body weight has been shown to be well-tolerated with little to no toxicity in mice [25].

### 2.5. Disease Activity Index (DAI) Score

The mice were checked daily for colitis based on their body weight, stool consistency, and gross rectal bleeding. The disease activity index (DAI) score was determined by combining scores of body weight, stool consistency, and gross rectal bleeding, as previously described [34]. Each score was measured as follows: body weight (0 = no change; 1 = 1–5%; 2 = 5–10%; 3 = 10–20% and 4 = ≥ 20%), stool consistency or diarrhea (0 = normal; 1 = some soft; 2 = soft; 3 = unformed/mild diarrhea and 4 = severe watery diarrhea), and visible fecal blood (0 = normal, 1 = slightly bloody; 2 = bloody; and 3 = blood in whole colon). Colons were immediately removed after sacrificing, washed with PBS then length of the colons was measured using caliper.

### 2.6. Histopathological Analysis

One-third of each colon was fixed with 4% paraformaldehyde. After dehydration and paraffin-embedding, colons were cut into 7 μm sections and stained with hematoxylin and eosin. The images of stained colon tissues were taken with a Moticam photomicroscope (Motic, San Antonio, TX, USA). Histological score was evaluated as described earlier [35,36]. The severity of histopathology was assessed based on the three following categories: epithelial changes (0: normal, 1: loss of epithelium structure, 2: erosion of epithelium, 3: severity destruction of epithelium), overall crypt damage (0: normal, 1: focal, 2: lesion of 1/3 area, 3: lesion of 2/3 area, 4: lesion of whole area) and quality and dimension of inflammatory cell infiltration (0: few infiltration of neutrophil, 1: neutrophil infiltration of less than 25% of sampled crypts, 2: neutrophil infiltration of more than 25% but less than 50% of sampled crypts, 3: neutrophil infiltration of more than 50% of sampled crypts).

### 2.7. Immunohistochemical Staining

Paraffin-embedded colon tissues were sectioned to 5 μm, then the sections were deparaffinized with xylene and rehydrated with ethanol. The sections were incubated in PBS containing 3% H_2_O_2_ for 10 min to inhibit endogenous peroxidase. The sections were then rinsed with PBS, and blocked with 1% bovine serum albumin for 30 min at room temperature. Then, sections were incubated with the inducible nitric oxide synthase (iNOS) antibody (Santa Cruz Biotechnology; dilution 1:100) at 4 °C overnight. After washing three times in PBS for 5 min each time, sections were incubated with secondary antibody (1:100) for 1 h at room temperature. The sections were stained by DAB chromogen kit and counterstained with hematoxylin.

### 2.8. Myeloperoxidase (MPO) Activity Assay

MPO activity was determined according to the manufacturer’s instructions for MPO activity assay kit (Abcam, Danvers, MA, USA). Briefly, colon samples (20 mg) were thawed and homogenized with cold assay buffer and then centrifuged at 20,000× *g* for 2 min at 4 °C. Fifty microliters of the supernatant were added to a black 96-well microplate with clear bottom and 50 μL of MPO reaction mixture was added. Fluorescence intensity was measured on a microplate reader at Ex/Em = 485/525 nm. All experiments were performed in triplicate. MPO activity was indicated as units/mg of protein.

### 2.9. Measurement of Cytokines and Chemokines in Serum

The levels of cytokines and chemokines in serum were determined using a Mouse XL Cytokine Array Kit (R&D, Minneapolis, MN, USA) following the manufacturer’s instructions. Briefly, membranes were blocked with blocking buffer for 1 h, serum samples were subsequently added and incubated overnight at 4 °C. Membranes were then incubated at room temperature for 1 h after mixing with a detection antibody cocktail. After washing, membranes were incubated for 30 min with streptavidin-horseradish peroxidase conjugate working solution. After washing, membranes were incubated for 1 min using a Chemi Reagent Mix, then exposed to an X-ray film for 5 min. Quantitative analysis of cytokine or chemokine spots were performed using a CS analyzer ver. 3.00 software (ATTO Co., Tokyo, Japan).

### 2.10. Western Blot

Colon tissues were homogenized with RIPA buffer containing a protease inhibitor and phosphatase inhibitor cocktail on ice. The procedure for Western blot has been described before [21]. The protein bands were captured using CCD camera system EZ-Capture II (ATTO & Rise Co., Tokyo, Japan) and data were analyzed by CS analyzer ver. 3.00 software (ATTO Co., Tokyo, Japan).

### 2.11. Statistical Analysis

Data were expressed as means ± standard deviations (SDs) of at least three independent experiments unless otherwise indicated. Data were analyzed using one-way analysis of variance (ANOVA), flowed by Tukey’s post hoc analysis. Differences with a value of *p* < 0.05 were considered statistically significant. All analyses were performed using SPSS for Windows, version 10.07 (SPSS Inc., Chicago, IL, USA).

## 3. Results

### 3.1. MES Ameliorated DSS-Induced Colitis

**Figure 2:**
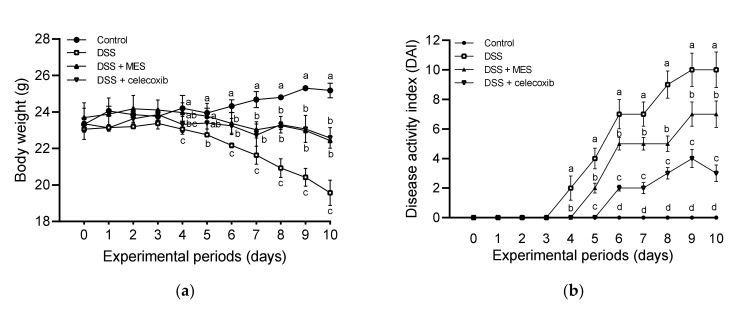

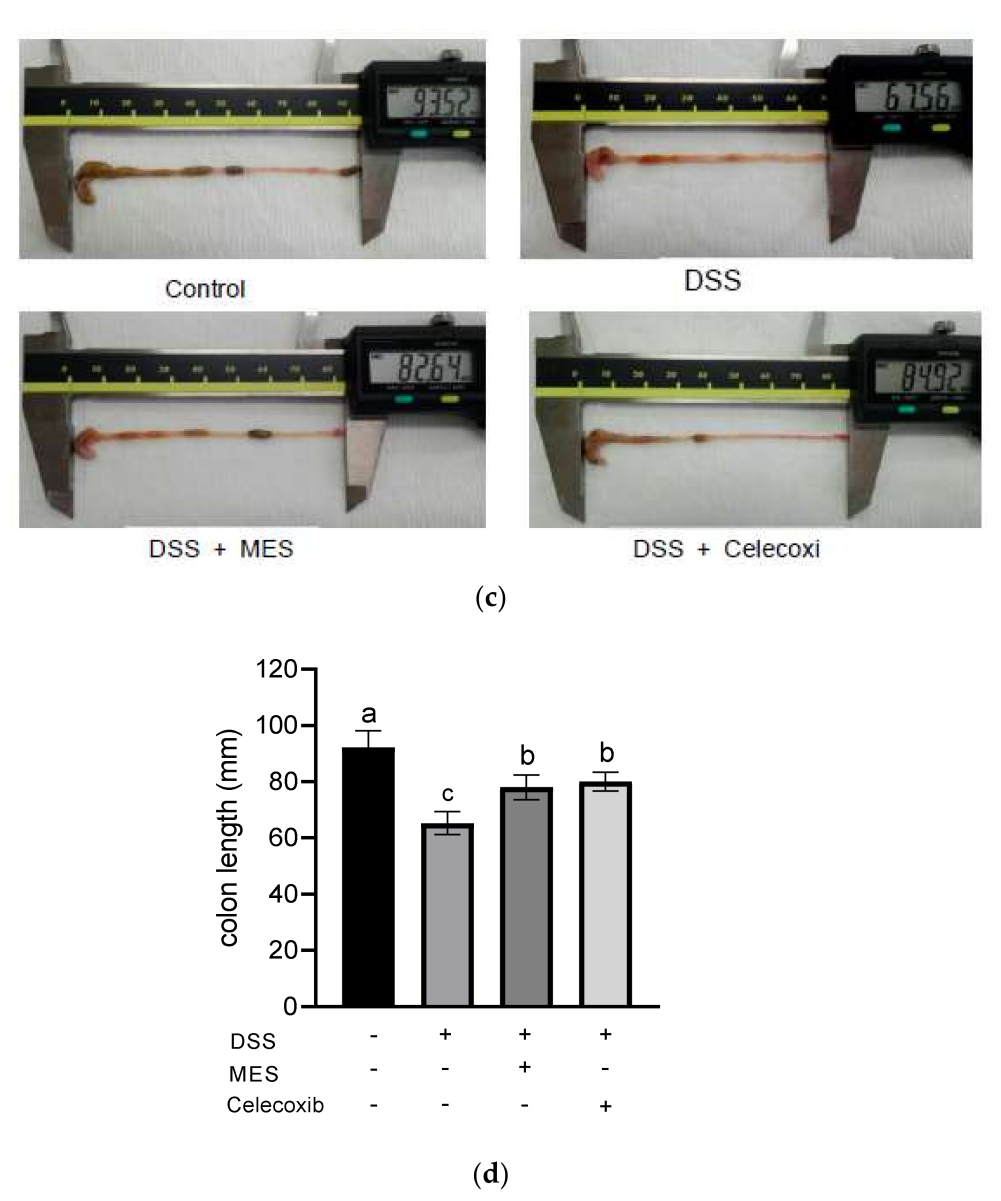
MES supplementation ameliorates symptoms of DSS-induced colitis. (**a**) Body weight from day 0 to day 10. (**b**) Disease activity index (DAI) scores. (**c**) Gross picture of colons, measured in mm. (**d**) Quantification of length of colons from each group mice obtained on Day 11. All data are expressed as mean ± SD. All data are expressed as mean ± SD. Bars with no common letters are significantly different (*p* < 0.05). DSS: dextran sulfate sodium; MES: meroterpenoid-rich ethanoic extract of a brown algae, *Sargassum macrocarpum*.

The protective effect of MES against colitis was evaluated by body weight, DAI, and colon length. As shown in Figure 2a, from Day 4 of the intervention, mice showed a significant body weight loss due to DSS, however, MES and celecoxib could attenuate such a loss. Furthermore, from Day 4, notable poor stool formation and rectal bleeding were observed in DSS-treated mice, led to a significant increase in DAI score (Figure 2b). Those symptoms were observed from Day 5 of MES group and Day 6 of celecoxib group. Compared to the DSS group, MES and celecoxib groups showed lower DAI scores until the end of the treatment course. Colon length shortening is another feature of colitis induced by DSS. As shown in Figure 2c,d, DSS treated group showed shorter colon length compared to the control group, while MES and celecoxib supplementations significantly alleviated the shortening.

### 3.2. MES Ameliorated Histological Damage in Colon Tissue and Inhibited Infiltration of Inflammatory Cells

**Figure 3:**
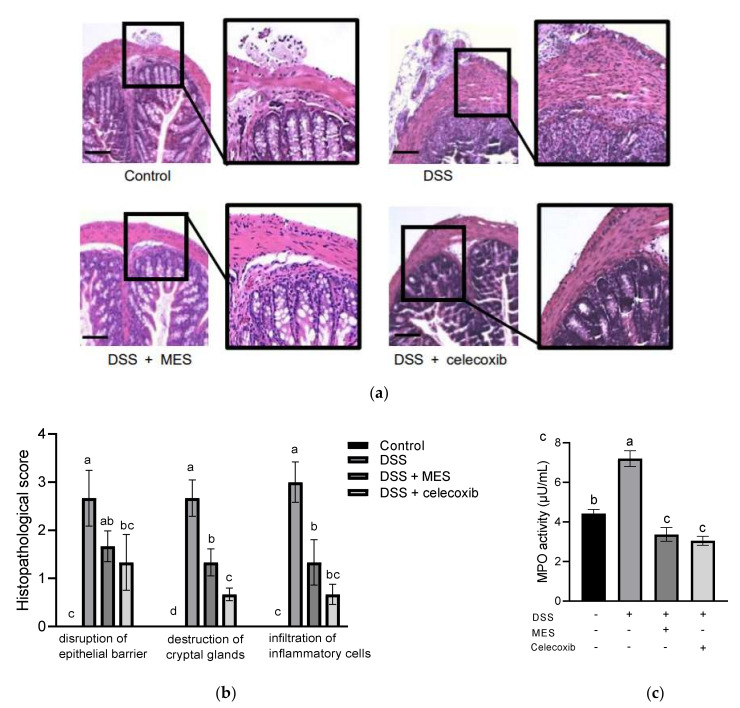
MES supplementation ameliorates DSS-induced tissue damage. (**a**) Hematoxylin and eosin (H & E) images (magnification, ×100 left), scale bar = 100 μm. (**b**) Histological scores. (**c**) Myeloperoxidase (MPO) activity of the colon tissues. All data are expressed as mean ± SD. Bars with no common letters are significantly different (*p* < 0.05). DSS: dextran sulfate sodium; MES: meroterpenoid-rich ethanoic extract of a brown algae, *Sargassum macrocarpum*.

DSS-treated mice exhibited pronounced histological changes, such as disruption of the epithelial barrier, destruction of cryptal glands structure, and infiltration of inflammatory cells (Figure 3a). MES and celecoxib supplementations significantly ameliorated these damages. In addition, quantified histological scores from MES and celecoxib groups were significantly lower than those of the DSS group (Figure 3b).

To examine the infiltration of inflammatory cells into the colon, MPO activity of colon tissue was also measured. As shown in Figure 3c, MPO activity was remarkably increased by DSS. Whereas MES and celecoxib strongly inhibited its activity, to an extent even lower than the control group. These results demonstrated that MES efficiently inhibits neutrophil infiltration into DSS-caused inflamed colon.

### 3.3. MES Suppressed the Release of Inflammatory Molecules in Colon Tissue and Serum

**Figure 4:**
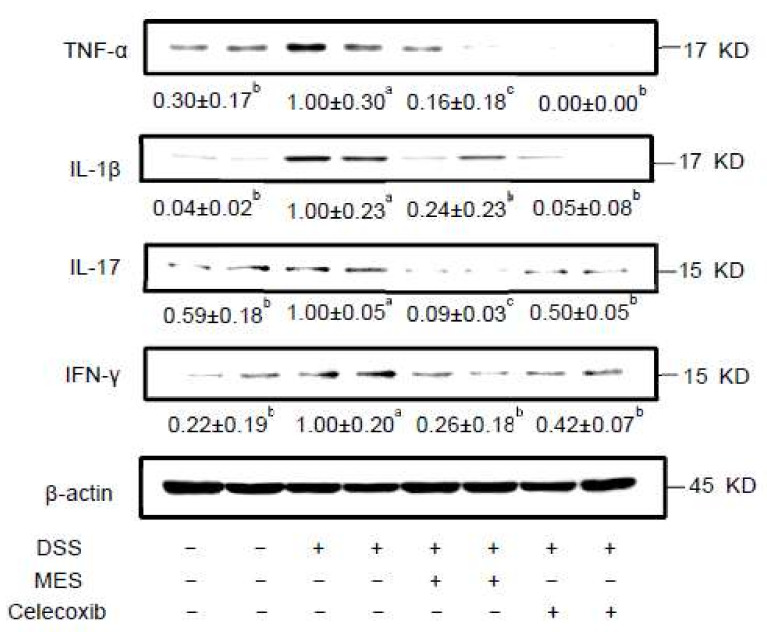
MES supplementation suppresses levels of pro-inflammatory cytokines in colon of DSS induced colitis mice. Protein levels of TNF-α, IL-1β, IL-17 and IFN-γ were determined by Western Blot. All data are expressed as mean ± SD. Bars with no common letters are significantly different (*p* < 0.05). DSS: dextran sulfate sodium; MES: meroterpenoid-rich ethanoic extract of a brown algae, *Sargassum macrocarpum*.

DSS markedly elevated the level of pro-inflammatory cytokines such as TNF-α, IL-1β, IL-17, and IFN-γ in colon (Figure 4). Meanwhile, MES treatment strongly suppressed the elevation of these inflammatory mediators. These results suggest that the protective effect of MES on DSS-induced colitis is largely related to the suppression of pro-inflammatory cytokines.

**Figure 5:**
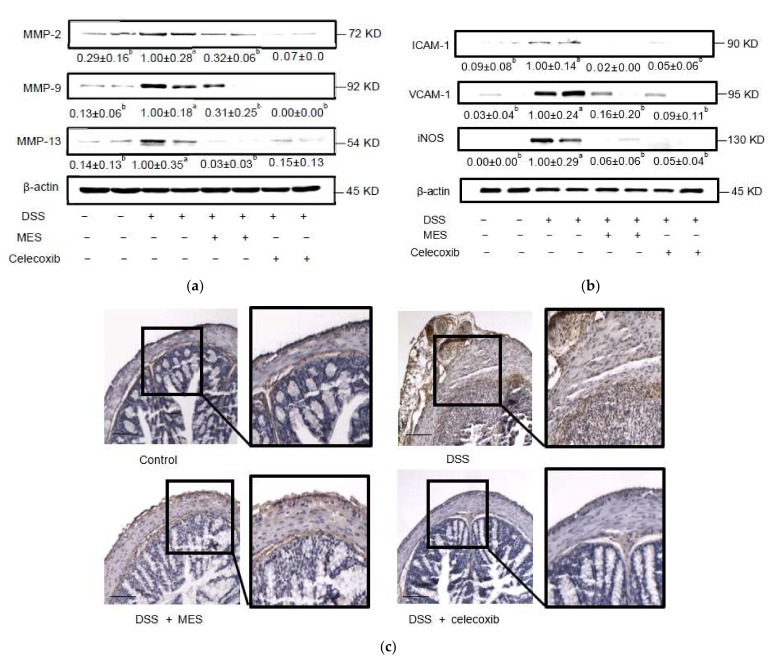
MES supplementation represses levels of inflammation-related proteins in colon of DSS-induced colitis mice. Total protein levels of (**a**) MMP-2, -9, and -13 and (**b**) ICAM-1, VCAM-1, and iNOS were determined by Western Blot. (**c**) Representative immunohistochemically stained slides with iNOS antibody in colon tissues (magnification, ×100 left), scale bar = 100 μm. All data are expressed as mean ± SD. Bars with no common letters are significantly different (*p* < 0.05). DSS: dextran sulfate sodium; MES: meroterpenoid-rich ethanoic extract of a brown algae, *Sargassum macrocarpum*; MMP, matrix metalloproteinase; ICAM-1, intercellular adhesion molecule-1; VCAM-1: vascular cell adhesion molecule-1; iNOS, inducible nitric oxide synthase.

The overexpression of inflammatory-related proteins, MMP-2, MMP-9 and MMP-13 has been reported to be involved in colon damage during colitis [37]. DSS exposure significantly elevated MMP-2, MMP-9 and MMP-13 expressions in colon, however, MES and celecoxib strongly attenuated their production (Figure 5a).

Adhesion molecules, such as ICAM-1 and VCAM-1, suppress the circulation of lymphocytes and propagate the inflammatory process by stimulating their migration into inflamed bowel tissue. DSS induced the expression of ICAM-1 and VCAM-1 in colon, which was strongly inhibited by MES (Figure 5b). Besides ICAM-1 and VCAM-1, iNOS expression was also strongly attenuated by MES administration compared to the DSS-treated mice. As shown in Figure 5c, the reduced expression of iNOS in colonic mucosa by MES and celecoxib was corroborated by immunohistochemical analysis. However, the difference was less pronounced than Western blot. It may be due to the nonspecific binding in the immunohistochemical samples.

Besides the inflammatory status of the colon, the level of pro-inflammatory cytokines and chemokines in serum was also assessed using a Proteome Profiler Array assay kit. As shown in Supplementary Figure S1a,b, DSS increased the level of a variety of chemokines (MCP-1, RANTES, MIP-3β, MIP-1α, KC, and MIP-2), inflammatory molecules (myeloperoxidase, E-selectin, and P-selectin), and ILs. Meanwhile, MES and celecoxib suppressed the elevation of those pro-inflammatory molecules. The cytokines (MMP2, MMP-9, IL-17A and IFN-γ) suppressed by MES in the colon was also lower in the serum of MES group. However, levels of TNF-α and IL-1β were suppressed by MES in colon but not in serum. In addition, MES and celecoxib also showed different effects on the release of cytokines, such as MIP-1α/β, CD14, or IL-23.

### 3.4. MES Suppressed Signaling Proteins in Colon Tissue

**Figure 6:**
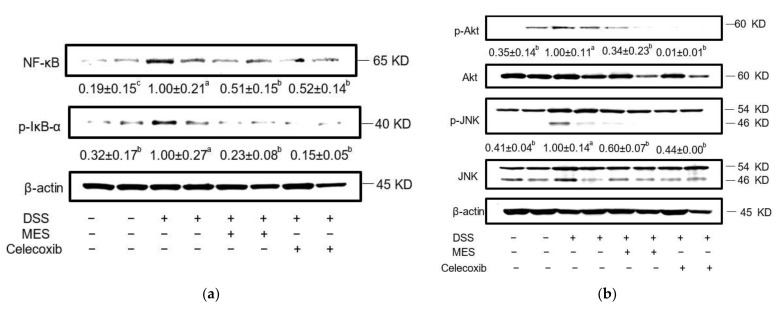
MES administration inhibits NF-κB, Akt and JNK signal pathways in DSS-induced colon tissues. Protein levels of (**a**) NF-κB, p-IκBα, (**b**) p-Akt, Akt, p-JNK, and JNK were determined by Western Blot. All data are expressed as mean ± SD. Bars with no common letters are significantly different (*p* < 0.05). DSS: dextran sulfate sodium; MES: meroterpenoid-rich ethanoic extract of a brown algae, *Sargassum macrocarpum*; NF-κB, nuclear factor kappa-light-chain-enhancer of activated B cells; Akt, protein kinase B; JNK, Jun N-terminal kinase.

NF-κB regulates the expression of pro-inflammatory cytokines and mediators in inflammatory responses [38]. The activation of NF-κB is primarily regulated by interaction with inhibitory IκB proteins. As shown in Figure 6a, in colon tissue, DSS strongly elevated the phosphorylation of NF-κB (p65), while MES administration markedly suppressed its phosphorylation. In line with that, DSS induced the phosphorylation of IκB-α, an inhibitor of NF-κB activation, which was not seen in the MES and celecoxib groups.

MAPKs (e.g., ERK, JNK and p38 MAPK) and Akt have been implicated in the regulation and release of pro-inflammatory cytokines in the course of UC [26,39]. DSS treatment resulted in a marked increase of the phosphorylation of Akt and JNK (Figure 6b), but not ERK and p38 MAPK (data not shown). Treatment with MES and celecoxib strongly suppressed the phosphorylation of Akt and JNK.

These results indicated that MES inhibited the production of pro-inflammatory cytokines through blocking of the phosphorylation of Akt, JNK and NF-kB signaling pathways in DSS-induced colitis.

## 4. Discussion

The majority of colitis patients suffer from a chronic disease course with intensive pain, rapid weight loss, swelling of the colon tissue, and rectal bleeding [1]. Treatments for colitis include TNF–α antagonist, aminosalicylates, steroids, and immunomodulators [8]. However, adverse effects of these drugs, such as gastrointestinal intolerance, hepatotoxicity and increased malignancies, make it necessary to develop effective and safe alternative treatment [40,41].

MES, a meroterpeniod rich extract of brown alga *S. macrocarpum*, exhibited a strong anti-inflammatory activity, in multiple cell and animal models [24,33,42]. Its anti-inflammatory and immune-modulating activity is attributable to a panel of components including SHQA, SCM and SQA [21,32,43,44,45]. In this study, we aimed to assess the effect of MES in colitis, an inflammatory disease, based on a DSS-induced colitis mouse model. Our results show that administration of MES significantly ameliorate DSS-induced colitis via modulation of mucosal barrier and inflammation.

DSS-fed mice present distinct symptoms of colitis, including body weight loss, colon shortening, bloody diarrhea, disruption of epithelial barrier, destruction of crypt, and infiltration of inflammatory cells [46]. Our results demonstrate that many of the symptoms were effectively alleviated by the supplementation of MES. The effect of MES at 12 mg/kg was comparable to celecoxib at 10 mg/kg. As a selective inhibitor of COX-2, celecoxib is prescribed for the management of pain and inflammatory disorders [29,47], and was used as a positive control in the present study.

Defects of epithelial barrier and cryptal glands are strongly implicated in the pathogenesis of colitis, since disruption of barrier integrity facilitates the translocation of lumen bacteria into the mucosa [47,48]. Infiltration of immune cells into the intestinal mucosa in response to insult in the colon is another key feature of colitis [49]. Based on histological images, the lesion of colon and inflammatory cell infiltration in DSS-induced mice was ameliorated by MES to an extent similar with celecoxib. MPO is a heme-containing peroxidase released at sites of inflammation by activated leukocytes and excessive generation of MPO-derived oxidants have been linked to tissue damage in IBD [50]. Our results revealed that increased MPO activity in DSS mice was effectively suppressed by both MES and celecoxib. Overall, our findings support a protective role of MES against tissue damage in colitis.

Pro-inflammatory cytokines produced by hyperactivated immune and stromal cells are pivotal in the initiation and development of colitis. The imbalance between pro-inflammatory and anti-inflammatory cytokines in IBD leads to disease progression and tissue destruction [51]. For instance, TNF-α induces hypervascularization and angiogenesis, causes barrier alterations and promotes cell death of intestinal epithelial cells [51]. IL-1β promotes colitis-associated tumorigenesis [52]. Deficiency of those pro-inflammatory cytokines results in reduced colitis and anti-cytokine therapies involving TNF-specific agents composes an important part of clinical therapy in IBD treatment [53,54,55,56]. In MES and celecoxib groups, we observed lower levels of pro-inflammatory cytokines, including interferon-γ (IFN-γ), TNF-α, IL-1β, and IL-17 in colon biopsies. This was in line with the decreased immune cell infiltration into colon. The serum cytokine array results also corroborated such an anti-inflammatory function of MES and celecoxib. The ability of seaweed to regulate imbalance immune system in intestinal inflammation has been reported. For example, the administration of Laminaria japonica strongly inhibited pro-inflammatory cytokines in DSS-induced colitis mice [16].

Matrix metalloproteinases (MMPs) comprise a large group of zinc- and calcium-dependent endopeptidases, which are involved in the degradation of extracellular matrix components, including collagen, glycoproteins and proteoglycans, and cleavage of bioactive proteins [37]. MMP expression increases in inflamed intestinal tissue of IBD patients, and the symptoms of acute colitis could be attenuated by synthetic MMP inhibitors or MMP-9 knockout [57,58,59]. The role of MMPs in IBD may be contributed to their regulation on epithelial barrier function, immune response, angiogenesis and fibrosis [12,60]. In this study, we observed that the elevation of MMP-2, -9, and -13 expressions in colon of DSS-treated mice was markedly inhibit by MES as well as celecoxib.

The expression of adhesion molecules such as ICAM-1 and VCAM-1 are upregulated by cytokines and they function to stop the circulation of lymphocytes and allow their migration into inflamed bowel tissue and propagate the inflammatory process [11,61]. Anti-adhesion molecules have been developed for the management of IBD [11]. In the present study, downregulated expression of ICAM-1 and VCAM-1 in colon by MES administration in DSS-fed mice was observed. The lower ICAM-1 and VCAM-1 expression level was in line with the lower cytokine level of colon in MES group mice, and the attenuated expression of ICAM-1 and VCAM-1 in turn repressed the migration of leukocytes into colon.

Besides pro-inflammatory cytokines, inducible iNOS is another inflammatory marker whose expression is predominantly at the site of inflammation [62]. Overproduction of nitric oxide by iNOS has been implicated in colitis, and iNOS deficient mice showed reduced sensitivity to DSS-induced colitis [62]. Along with alleviated levels of pro-inflammatory cytokines, MMPs, and adhesion molecules, MES-induced diminished iNOS level supported a strong anti-inflammatory capacity of MES in colitis.

One major pro-inflammatory transcription factor, NF-κB, is markedly activated in IBD patients and strongly influences the course of mucosal inflammation [63]. In unstimulated cells, NF-κB is retained in the cytoplasm by NF-κB-inhibitor of kappa B (IκB)-α. Upon activation, IκB-α is phosphorylated and detached from NF-κB, which leads to the translocation of NF-κB into the nucleus, where it binds to downstream target gene DNA binding site [64]. Effects of the therapeutic drugs used to treat IBD such as anti-TNF-α antibodies, sulfasalazine and methotrexate are known related to suppression of NF-κB activity [24]. Previous studies have shown that some *Sargassum* species exert immune response regulation function through NF-κB signaling pathway in vivo and in vitro [65,66,67,68]. Our data revealed that MES administration suppressed the expression of NF-κB and the phosphorylation of IκB-α. The ability of MES and its active components SHQA and SQA to inhibit NF-κB activation was also seen in various cell models and collagen-induced rheumatoid arthritis mice [20,21,24,25].

Akt and JNK signaling pathways are also implicated in the regulation and release of pro-inflammatory cytokines, and promote the progress of colitis [26,39]. The protective role of Akt and JNK inhibitors on colitis have been reported [69,70]. Our results indicated that phosphorylation of Akt and JNK were elevated in colon of colitis mice, however MES supplementation strongly suppressed their activation. The inhibition of MES on Akt and NF-κB may be contributed to its active compounds SHQA and SQA [20,21,23,71].

This study also has its limitations. For example, functional evaluation of intestinal permeability was not performed; BALB/c mice were used in inducing acute colitis, instead of strains that could develop chronic colitis after induction, such as C57BL/6; whether the beneficial effects was due to the treatment effect of MES or due to its interference of induction of colitis was not investigated in this study; IBD is a chronic inflammatory condition, while an acute colitis model was used in current study, the effect of MES on human UC needs to be further studied.

## 5. Conclusions

In conclusion, our study showed the administration of MES at 12 mg/kg for 10 days significantly alleviated symptoms of colitis induced by DSS, and suppressed the corresponding infiltration of inflammatory cells, production of pro-inflammatory cytokines, expression of adhesion molecules and inflammatory enzymes. Mechanism exploration suggests that MES may exert those functions through inhibiting NF-κB, Akt, and JNK signaling pathways.

## Data Availability

The datasets generated for this study are available on request to the corresponding author.

## Supplementary Materials

The following supporting information can be downloaded at: https://www.mdpi.com/article/10.3390/foods11030329/s1, Figure S1: MES supplementation suppresses levels of pro-inflammatory cytokines in serum of DSS-treated mice. (a). Proteome Profile Mouse XL Cytokine Array kit was used to semi-quantify mouse cytokines in the pooled serum from each group. (b). Heatmap analysis illustrated the spot density of cytokines suppressed by MES or celecoxib.
