# Supplementary material for: Meroterpenoid-Rich Ethanoic Extract of Sargassum macrocarpum Ameliorates Dextran Sulfate Sodium-Induced Colitis in Mice
Source: Foods. 2022 Jan 25;11(3):329. doi: 10.3390/foods11030329 (PMC8834051; doi:10.3390/foods11030329)
Supplement: Supplementary file 1 [file foods-11-00329-s001.zip › foods-1547639-supplementary.pdf]

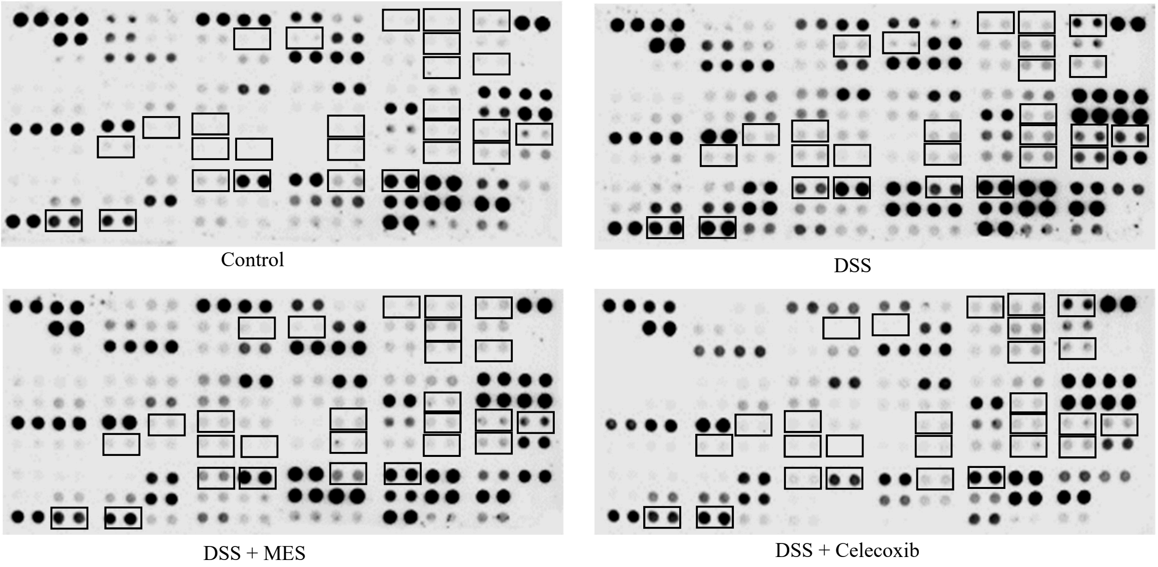

(a)

	Control	DSS	DSS+MES	DSS+celecoxib
MCP-1	0.357	1	0.37	0.143
MIP-1 α/β	0.12	1	0.358	1.299
RANTES	0.146	1	0.071	0.211
MIP-3β	0.104	1	0.551	2.00E-03
MIP-3α	0.266	1	0.363	0.014
CD14	0.211	1	0.649	1.077
KC	0.114	1	0.392	0.619
MIP-2	0.182	1	0.612	0.747
IFN-γ	0.195	1	0.04	0.487
M-CSF	0.343	1	0.597	0.216
MMP-2	0.691	1	0.03	0.293
MMP-9	0.393	1	0.552	0.256
Myeloperoxidase	0.565	1	0.429	0.361
E-selectin	0.498	1	0.504	0.413
P-selectin	0.27	1	0.38	0.415
IL-1α	0.191	1	0.688	0.334
IL-1β	0.193	1	0.902	0.263
IL-3	0.158	1	0.723	0.346
IL-5	0.16	1	0.415	0.397
IL-6	0.121	1	0.49	0.294
IL-7	0.253	1	0.645	0.346
IL-12p40	0.407	1	0.504	0.301
IL-15	0.07	1	0.653	0.119
IL-17A	0.668	1	0.505	0
IL-23	0.028	1	0.932	0.032
IL-28A/B	0.253	1	0.331	0.324
IL-33	0.19	1	0.247	0.254

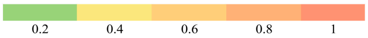

(b)

Supplementary Figure S1. MES supplementation suppresses levels of pro-inflammatory cytokines in serum of DSS-

treated mice. (a). Proteome Profile Mouse XL Cytokine Array kit was used to semi-quantify mouse cytokines in the pooled serum from each group. (b). Heatmap analysis illustrated the spot density of cytokines suppressed by MES or celecoxib.
